# Delamination and Skin-Spar Debond Detection in Composite Structures Using the Inverse Finite Element Method

**DOI:** 10.3390/ma16051969

**Published:** 2023-02-28

**Authors:** Rinto Roy, Marco Gherlone

**Affiliations:** Politecnico di Torino, Department of Mechanical and Aerospace Engineering, Corso Duca degli Abruzzi 24, 10129 Torino, Italy

**Keywords:** shape sensing, carbon fiber-reinforced polymer, composite plate, delamination detection, fiber optics, inverse problem

## Abstract

This work presents a novel strategy for detecting and localizing intra- or inter-laminar damages in composite structures using surface-instrumented strain sensors. It is based on the real-time reconstruction of structural displacements using the inverse Finite Element Method (iFEM). The iFEM reconstructed displacements or strains are post-processed or ‘smoothed’ to establish a real-time healthy structural baseline. As damage diagnosis is based on comparing damaged and healthy data obtained using the iFEM, no prior data or information regarding the healthy state of the structure is required. The approach is applied numerically on two carbon fiber-reinforced epoxy composite structures: for delamination detection in a thin plate, and skin-spar debond detection in a wing box. The influence of measurement noise and sensor locations on damage detection is also investigated. The results demonstrate that the proposed approach is reliable and robust but requires strain sensors proximal to the damage site to ensure accurate predictions.

## 1. Introduction

In recent decades, carbon fiber-reinforced polymer (CFRP) composites have emerged as promising alternatives to metallic constructions, especially in weight-sensitive applications like aircraft. Their high specific stiffness and strength guarantee low structural weight, leading to reduced fuel consumption, lower operational costs, and low environmental impact. Additionally, their superior durability, corrosion, and fatigue properties lead to low maintenance requirements [1]. However, a key limitation of composites is their failure and fatigue behavior, which are quite different and more complex than in metals [2,3]. The damage mechanisms in composites depend on the matrix and fiber materials used, composite layup, loading conditions, and environmental exposure [4]. Among the environmental factors, moisture absorption by the epoxy resin or at the fiber-matrix interface leads to a degradation of mechanical strength and integrity [1]. Similar degradation is also seen when CFRP composites are exposed to elevated temperatures due to the oxidation of the carbon fiber and resin decomposition. These effects are further exacerbated by the presence of sustained cyclic loads [5]. Finally, low-velocity impacts can also lead to internal delamination with barely any visible marks on the structural surface, thus going undetected. In this context, efficient structural health monitoring (SHM) of aircraft structures can identify such damage before they lead to catastrophic failures. SHM systems process data measured by a network of sensors mounted on the structure (or even embedded in the case of composites) to make real-time predictions of structural integrity [6]. SHM introduces a more condition-based maintenance (CBM) philosophy whereby structural integrity is assessed during operation, and maintenance activities are only executed when there is a potential risk to the structure. Such a maintenance approach reduces costs, lowers human effort, and improves overall structural safety [7].

Small and efficient sensing technologies are a key ingredient for SHM, and fiber optic strain sensors play a key role in these developments. They offer small size, flexibility, high sensitivity, durability, and high measurement density and can play an important role in real-time aircraft monitoring [8]. Damage or delamination detection based on fiber Bragg grating (FBG) or distributed fiber optic sensors have been an active area of research [9], some of which include the use of pattern recognition techniques to analyze the measured strain field [10], strain mapping for global monitoring [11], and measuring the dynamic strain profile for capturing changes in the strain mode shapes due to damage [12]. Efforts have also used distributed sensing for skin-stinger debond detection [13], and impact area estimation in composites [14]. Recently, self-sensing composites with embedded carbon nanotubes for monitoring strains have also emerged [15]. However, a key limitation of strain-based approaches is that strain changes due to damage are highly localized, requiring a high-density strain sensor network for accurate results [11].

The inverse problem of ’shape sensing’ presents an interesting opportunity to develop systems that obey such a real-time monitoring paradigm [16]. Shape-sensing methods can reconstruct the structure’s continuous displacement field using discrete strain measurements in real-time. Furthermore, these displacement results can be used to compute the strain or stress fields, forming the basis of an efficient SHM system for damage detection, prognosis, and fatigue monitoring. Existing literature on shape sensing is vast [16], with the use of basis functions [17,18] and integrating the measured strains [19,20] forming some interesting solution approaches. However, in recent years, shape sensing based on a variational principle has perhaps found the greatest acceptance. Work on this topic was pioneered by Tessler and Spangler [21,22], who proposed the inverse Finite Element Method (iFEM) for the shape sensing of plate or shell structures.

iFEM is a variationally-based approach that uses the finite-element-discretization framework and the strain-displacement relations for shape sensing [22]. Initial work by Tessler et al. [23] focused on the development of two-dimensional (2D) inverse elements based on Mindlin theory [24] for monitoring plates or shells, such as the three-node shell iMIN3. Over the years, additional higher-order elements for shells [25,26] and multi-layered composite or sandwich structures [27,28] have been developed. These development have also led to its application for various metallic and composite structures. Some of these efforts include the deformation monitoring of wing-like geometries [29], helicopter tail panels [30], stiffened panels [31,32], and wing boxes [33]. Experimental investigations include the shape sensing of a wing-shaped sandwich structure [34], and a carbon fiber reinforced polymer (CFRP) and aluminum honeycomb sandwich panel using distributed fiber-optic sensors [35].

The success of iFEM has also inspired SHM methodologies based on it [36]. They have been applied for detecting damages in beams [37,38], thin plates [39,40], aircraft wings [41], and monitoring offshore wind turbine structures [42,43]. Approaches to characterize the damage include coupling iFEM with convolutional neural networks for detection and localization [44], peridynamics for crack propagation monitoring [45], and physics-based pre-extrapolation for reconstructing displacements and strains around discontinuities [46]. iFEM has also been demonstrated for damage monitoring in composite structures [47,48,49]. Specifically, Colombo et al. [48] applied iFEM for detecting impact damages in composite panels under compressive loads, while Kefal et al. [49] investigated intra-lamina damage detection using surface and embedded sensors. This latter work used iFEM-based strain sensing in conjunction with a priori healthy baseline for detecting damage. Despite the promise of these efforts, they are also plagued by certain limitations. Although damage detection approaches usually rely on data from a system model or healthy state of the structure, such information is rarely available [49]. Also, in the case of composites, embedding sensor within the structure presents its own difficulties, while the need for excessive experimental strain measurements [48] is another limitation.

This work aims to combine recent advances in distributed fiber optic sensors with the capabilities of the iFEM for displacement sensing using surface strain measurements to propose a novel damage detection and fatigue monitoring strategy for composite structures. Rather than a system model or a priori sensor measurements of the healthy structure, this work uses a dynamic baseline estimated in real-time from the iFEM results to formulate a damage index. As the iFEM is inherently independent of the structure’s material properties or operational conditions, the use of a real-time baseline further improves the practical viability of the strategy. This work also attempts to assess the reliability and robustness of the method by investigating the influence of structural operational conditions, measurement noise, sensor configurations used, geometrical complexity of the structure, and type of composite failure on damage diagnosis. A key challenge of composites is their numerous intra- and inter-lamina failure modes. Hence, two damage scenarios are investigated in this work: delamination in vibrating composite plates and skin-spar debonding in wing boxes. Such a comprehensive investigation aims to provide a clear delineation between the achievements and limitations of the method, paving the way for future efforts and developments.

The paper is organized as follows. The theoretical formulation of the 2D iFEM and the methodology used to establish a healthy baseline is briefly discussed in Section 2. Section 3 presents a numerical study on a vibrating composite plate, where the proposed approach is applied for delamination detection. A comprehensive investigation of the various parameters that influence damage detection is presented, with results demonstrating the accuracy and robustness of the method, specifically for damage close to the plate surface or when excited at high frequencies. These conclusions are further evaluated in Section 4 where the method is applied for debond detection in a composite wing box. This section is also promising, highlighting the ability of the method in monitoring geometrically-complex structures and various composite failure scenarios. Finally, Section 5 concludes with the main achievements and limitations of the research and topics for future work.

## 2. Methodology

The theoretical formulation of the 2D iFEM, originally proposed by Tessler and Spangler [22], is briefly recounted here. Subsequently, the smoothing methodology used to establish a structural baseline from the iFEM results is also discussed. Finally, details of the damage detection strategy are presented.

### 2.1. Inverse Finite Element Method

**Figure 1:**
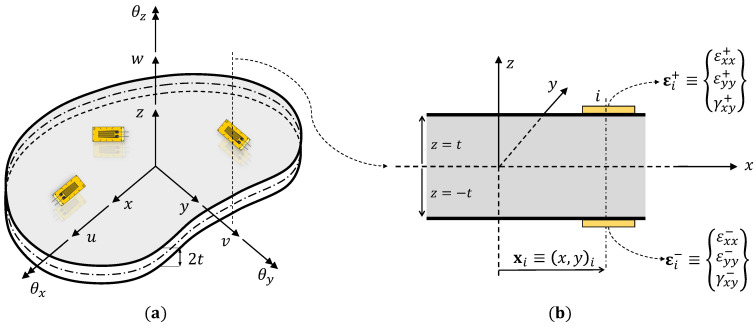
Illustration of the plate structure: (**a**) kinematic variables used to represent the deformations, and (**b**) strain sensors instrumented on the top and bottom surfaces of the plate.

Considering a plate or shell structure defined in the three-dimensional (3D) Cartesian coordinate frame $\left(x,y,z\right)\subset R^{3}$ (shown in Figure 1). The plate mid-plane is defined by the orthogonal coordinates, x≡(x,y), with plane normal along the *z*-axis. The plate has a thickness 2t (where z∈[−t,t]), and a mid-plane area *A* (located at z=0).

#### 2.1.1. Mindlin Plate Kinematics

The 2D iFEM for plates or shells is based on the kinematic assumptions of Mindlin theory [24]. The Cartesian components of the displacement vector can be represented in terms of the kinematic variables, $u\equiv {\left\{u,v,w,\theta_{x},\theta_{y}\right\}}^{T}$, as

(1) $$u_{x}=u\left(x\right)+z\theta_{y}\left(x\right)\;,\;u_{y}=v\left(x\right)-z\theta_{x}\left(x\right)\;,\;u_{z}=w\left(x\right)$$

where $u_{x}$, $u_{y}$, and $u_{z}$ are the displacements of any plate point in the *x*, *y*, and *z*-directions, respectively. The kinematic variables, *u* and *v*, are the mid-plane surface displacements in the *x* and *y*-directions; *w* is the transverse deflection averaged across the plate thickness; and $\theta_{x}$ and $\theta_{y}$ are the rotations of the section normal about the *x* and *y*-axes, respectively. The kinematic variables of the plate are shown in Figure 1.

Using Equation (1), the strain field is computed from the linear strain-displace- ment relations:

(2) $$\left\{\begin{array}{c} \epsilon_{xx}\\ \epsilon_{yy}\\ \gamma_{xy} \end{array}\right\}=\left\{\begin{array}{c} u_{x,x}\\ u_{y,y}\\ u_{x,y}+u_{y,x} \end{array}\right\}=\left\{\begin{array}{c} u_{,x}\\ v_{,y}\\ u_{,y}+v_{,x} \end{array}\right\}+z\left\{\begin{array}{c} \theta_{y,x}\\ -\theta_{x,y}\\ -\theta_{x,x}+\theta_{y,y} \end{array}\right\}=e\left(u\right)+zk\left(u\right)$$

where strain measures, e(u) and k(u), represent the in-plane stretching and curvature of the plate mid-plane. The transverse shear strains based on Mindlin theory are given as

(3) $$\left\{\begin{array}{c} \gamma_{xz}\\ \gamma_{yz} \end{array}\right\}=\left\{\begin{array}{c} u_{z,x}+u_{x,z}\\ u_{z,y}+u_{y,z} \end{array}\right\}=\left\{\begin{array}{c} w_{,x}+\theta_{y}\\ w_{,y}-\theta_{x} \end{array}\right\}=g\left(u\right)$$

where g(u) are the transverse shear strain measures of the plate. These eight strain measures, e, k, and g, form the analytical part of the 2D iFEM error functional.

#### 2.1.2. Experimental Strain Measures

The plate is assumed to be instrumented with strain sensors (strain gauges or fiber optics) at *N* discrete locations $x_{i}={\left(x,y\right)}_{i}$, where i=1,...,N (see Figure 1). The sensors are mounted both on the top, z=t, and bottom, z=−t, surfaces of the plate, measuring strains $\varepsilon_{i}^{+}={\left\{\varepsilon_{xx}^{+},\varepsilon_{yy}^{+},\gamma_{xy}^{+}\right\}}_{i}^{T}$ and $\varepsilon_{i}^{-}={\left\{\varepsilon_{xx}^{-},\varepsilon_{yy}^{-},\gamma_{xy}^{-}\right\}}_{i}^{T}$, respectively. The strain measures in the reference mid-plane coordinates of the plate can be computed from the experimental strain measurements as

(4) $$e_{i}^{\epsilon }=\frac{1}{2}{\left(\left\{\begin{array}{c} \epsilon_{xx}^{+}\\ \epsilon_{yy}^{+}\\ \gamma_{xy}^{+} \end{array}\right\}+\left\{\begin{array}{c} \epsilon_{xx}^{-}\\ \epsilon_{yy}^{-}\\ \gamma_{xy}^{-} \end{array}\right\}\right)}_{i}\;,\;k_{i}^{\epsilon }=\frac{1}{2t}{\left(\left\{\begin{array}{c} \epsilon_{xx}^{+}\\ \epsilon_{yy}^{+}\\ \gamma_{xy}^{+} \end{array}\right\}-\left\{\begin{array}{c} \epsilon_{xx}^{-}\\ \epsilon_{yy}^{-}\\ \gamma_{xy}^{-} \end{array}\right\}\right)}_{i}$$

The transverse shear strains, $g^{\varepsilon }$, cannot be computed directly from experimental strains. However, based on the equilibrium equation, $g^{\varepsilon }$ can be computed indirectly from the first derivatives of $k^{\varepsilon }$. More details regarding this procedure are provided in Ref. [22].

#### 2.1.3. Least-Squares Error Functional

The 2D iFEM formulation is based on the finite element framework where the structural domain is discretized using a series of inverse finite elements with elemental areas, $A^{e}$. For each inverse element, *e*, a weighted least-squares error functional between the analytical and experimental strain measures is defined as

(5) $$\begin{array}{c} \Phi^{e}\left(u^{e}\right)\equiv w_{m}\Phi_{m}\left(u^{e}\right)+w_{b}\Phi_{b}\left(u^{e}\right)+w_{s}\Phi_{s}\left(u^{e}\right) \end{array}$$

where $u^{e}$ is the vector of nodal degrees-of-freedom (DOF) of each element, $\Phi_{m}$, $\Phi_{b}$, and $\Phi_{s}$ are the individual element error functionals based on the membrane, curvature, and transverse shear strain measures, respectively, and $w_{m}$, $w_{b}$, and $w_{s}$, are row vectors of weighting coefficients. The individual error functionals are written as

(6) $$\begin{array}{cc} \Phi_{m} & \equiv \left\{\begin{array}{c} \phi_{1}\\ \phi_{2}\\ \phi_{3} \end{array}\right\}=\frac{1}{A^{e}}\int_{A^{e}}{\left[e\left(u^{e}\right)-e^{\epsilon }\right]}^{2}dA\;,\;\Phi_{b}\equiv \left\{\begin{array}{c} \phi_{4}\\ \phi_{5}\\ \phi_{6} \end{array}\right\}=\frac{{\left(2t\right)}^{2}}{A^{e}}\int_{A^{e}}{\left[k\left(u^{e}\right)-k^{\epsilon }\right]}^{2}dA\\ \Phi_{s} & \equiv \left\{\begin{array}{c} \phi_{7}\\ \phi_{8} \end{array}\right\}=\frac{1}{A^{e}}\int_{A^{e}}{\left[g\left(u^{e}\right)-g^{\epsilon }\right]}^{2}dA \end{array}$$

The vectors of weighting coefficients control the degree of enforcement between the analytical and experimental sectional strains in the element error functional, thus influencing the individual element contribution to the global error functional. In cases where experimental strain measures are known, the weighting coefficients are set to unity ($w_{m}=w_{b}=\left\{1,1,1\right\}$ and $w_{s}=\left\{1,1\right\}$). However, as $g^{\epsilon }$ cannot be computed directly from experimental strains, the corresponding weighting coefficients are set to a small value ($w_{s}=\left\{10^{-5},10^{-5}\right\}$), and the squared norm has the following form:

(7) $$\begin{array}{c} \Phi_{s}\equiv \left\{\begin{array}{c} \phi_{7}\\ \phi_{8} \end{array}\right\}=\frac{1}{A^{e}}\int_{A^{e}}{\left[g\left(u^{e}\right)\right]}^{2}dA \end{array}$$

Equation (7) can be interpreted as a weak enforcement of the Kirchhoff (zero transverse shear) constraint. Hence, it is consistent with application to thin plates where the transverse shear deformations are much smaller than those due to the bending. The displacements and strains within an element are approximated by interpolating the nodal DOF using anisoparametric shape functions [25]. The iFEM solution involves minimizing the element functional of Equation (5) with respect to $u^{e}$, resulting in a set of linear algebraic equations. Assembling element contributions using the appropriate local-to-global coordinate transformations, the global matrices of the structure are obtained:

(8) KU=F

where the matrix K is only a function of the strain-sensor positions and F is a function of the sensor positions and measured experimental strains.

Similar to the direct FEM, the boundary conditions are applied to constrain against rigid-body motion and ensure a non-singular system matrix. Subsequently, K can be inverted, and Equation (8) solved to obtain the reconstructed nodal displacements, U. Once inverted, K does not need to be recomputed. The present work uses the four-node quadrilateral element iQS4 [25], with C^0^-continuous anisoparametric interpolations. For more details on the element formulation and the 2D iFEM, refer to Refs. [22,25].

As iFEM is based on the strain-displacement relations, it is independent of the material properties or operational conditions of the structure. Additionally, the adoption of the finite element discretization framework to model structures allows complex geometries to be modeled and analyzed in a computationally efficient manner. However, iFEM accuracy is influenced by the accuracy of the measured strains and the complexity of the strain distribution investigated. While measurement errors and environmental factors influence the former, the latter depends on the fidelity of the iFEM mesh in modeling the strains. Complex strain distributions require a high-fidelity mesh or higher-order inverse elements (with an associated increase in strain measurements required), increasing the computational cost of the analysis. iFEM is also influenced by the number and location of the strain measurements, and the boundary conditions applied. Conditions that could lead to a breakdown of the iFEM analysis include a lack of sufficient strain measurements to accurately describe the analyzed strain field (or the relevant strain components) or no measures at key locations of the structure (e.g., close to or in elements where the nodal DOF are constrained). Additionally, the absence of boundary conditions to constraint against the rigid-body motion also leads to erroneous iFEM results.

### 2.2. Smoothing Element Analysis

The presence of damage on a structure is usually assessed by comparing its current state to a healthy baseline. However, prior knowledge of a baseline requires access to an accurate system model or experimental data from the healthy structure, which is rarely available. The present work circumvents this limitation by computing a baseline from the iFEM results by smoothing local perturbations due to damage in the reconstructed displacements or strains. Although various smoothing methodologies exist in the literature (some are explored by Oboe et al. [50]), the smoothing element analysis (SEA) is used in the present work. The SEA is a variational method proposed for ‘smoothing’ finite element-derived non-smooth stress fields [51], allowing for more accurate recovery of boundary stresses and posterior error estimation. An improved SEA formulation [52], where an additional contribution due to second-order derivatives of the smoothed quantity is introduced in the variational statement to improve reliability, is discussed here. An interesting application in this regard was proposed by Kefal et al. [53] where the SEA was combined with the iFEM for the efficient shape sensing of composite structures.

The SEA is based on the finite-element framework where the 2D region of interest, Ω=x=(x,y), is discretized using smoothing finite elements (where $\Omega^{e}$ represents the domain of element *e*). The SEA can be used to smooth or interpolate scalar quantities, e.g., unidirectional strains $\varepsilon_{i}^{\varepsilon }$ measured within Ω, and compute a smoothed $C^{1}$-continuous field, $\varepsilon^{s}$, with $C^{0}$-continuous derivatives, $\delta_{x}^{s}$ and $\delta_{y}^{s}$. This is accomplished by minimizing a penalized-discrete-least-squares error functional that, for each element, is formulated as

(9) $$\begin{array}{cc} \Phi_{s}^{e}= & \frac{1}{n}\sum_{i=1}^{n}{\left(\varepsilon_{i}^{\varepsilon }-\varepsilon^{s}\left(x_{i}\right)\right)}^{2}+\alpha \int_{\Omega^{e}}\left[{\left(\varepsilon_{,x}^{s}-\delta_{x}^{s}\right)}^{2}+{\left(\varepsilon_{,y}^{s}-\delta_{y}^{s}\right)}^{2}\right]d\Omega \\ \; & +\beta \Omega^{e}\int_{\Omega^{e}}\left[{\left(\delta_{x,x}^{s}\right)}^{2}+{\left(\delta_{y,y}^{s}\right)}^{2}+\frac{1}{2}{\left(\delta_{x,y}^{s}+\delta_{y,x}^{s}\right)}^{2}\right]d\Omega \end{array}$$

where *n* is the total number of strain measurements per smoothing element, and α and β are dimensionless parameters that control the smoothing procedure. Anisoparametric shape functions are used to interpolate the nodal DOF of each smoothing element, with $\varepsilon^{s}$ interpolated quadratically and $\delta_{x}^{s}$ and $\delta_{y}^{s}$ linearly. Minimizing Equation (9) with respect to the nodal DOF leads to a set of linear algebraic equations for the element. The global system of equations is obtained by assembling contributions from all the elements. Subsequently, the equations can be solved to obtain the nodal components of the smoothed scalar field.

The first term of Equation (9) represents the squared error between the smoothed and measured strains, the second term is related to the first-order derivatives of the smoothed strains, while the third term controls the second-order derivatives of the smoothed strain field. Parameter, α, is used to control the continuity of $\varepsilon^{s}$ derivatives in the second term, i.e., $\varepsilon^{s}$ approaches $C^{1}$-continuity as the value of α tends to infinity. The optimal choice of α depends on the problem under consideration and is investigated further in Section 3. Similarly, β controls the severity of constraints on the second-order derivatives of $\varepsilon^{s}$ depending on the perceived accuracy of the measured strains, i.e., in the case of noisy measurements, a high value of β can be used to filter the data. In the present work, it is set to a small value ($\beta =10^{-5}$).

The SEA is a purely mathematical approach to smoothing, dependent only on the input data, element discretization, and parameter values used. Interested readers are encouraged to refer to Refs. [51,52] for further details or discussions on the SEA.

### 2.3. Damage Detection Using the iFEM and SEA

The combined iFEM-SEA damage detection strategy is described as follows:

- The iFEM is used to reconstruct the full-field structural displacements from discrete in-situ strain-sensor measurements;
- Equation (2) is used to compute the in-plane strains $e^{iFEM}$ and curvatures $k^{iFEM}$ from the reconstructed displacements;
- The SEA is used to smooth strain perturbations due to damage and establish a healthy baseline ($e^{SEA}$ and $k^{SEA}$) of the structure;
- The damaged and healthy strains are used to compute a damage index that effectively detects both the presence and location of damage in the structure.

A damage index, $I_{D}$, defined as the difference between the damaged and healthy state of the structure, is proposed:

(10) $$\begin{array}{c} I_{D}\equiv \left|\kappa_{p}^{iFEM}-\kappa_{p}^{SEA}\right| \end{array}$$

where $\kappa_{p}$ is the component of curvature associated with the primary bending direction of the structure. Alternatively, the principal or Von Mises curvatures can be used in more complex deformation scenarios. A curvature-based damage index is employed because structures primarily undergoing bending deformation are investigated in this work.

Additionally, a normalized damage index, $I_{D}^{n}\in \left[0,1\right]$, for assessing the structure under different load magnitudes or operational conditions is also used:

(11) $$\begin{array}{c} I_{D}^{n}=\frac{I_{D}-{\left(I_{D}\right)}_{min}}{{\left(I_{D}\right)}_{max}-{\left(I_{D}\right)}_{min}} \end{array}$$

where subscripts ${\left(*\right)}_{min}$ and ${\left(*\right)}_{max}$ are the minima and maxima of the damage index field.

At this stage, it is also useful to consider some of the merits and limitations of the proposed strategy. As the iFEM is used to reconstruct the damaged strain field, damage detection is independent of the material properties and operational conditions of the structure. Similarly, as the SEA is used to compute a real-time baseline, no prior data or measurements on the healthy structure are required. Given that SEA is a purely mathematical tool, it preserves the inherent advantages of the iFEM as well. However, as strain is the physical quantity measured, the instrumented sensors should measure strain perturbations due to the damage for the method to be effective. Hence, it can be considered a local monitoring strategy employed in cases where the sensors are located close to the damage site, or the damage causes strain perturbations that influence sensors placed far away. Finally, the values of hyper-parameters used to control the iFEM and SEA significantly affect the results obtained and should be selected appropriately. These effects are investigated in more detail in the following sections.

## 3. Numerical Study on a Composite Plate

This section numerically demonstrates the proposed iFEM-SEA strategy for detecting delamination in a cantilevered composite plate. Assuming the plate to be vibrating under cyclic loads, damage detection is assessed for the various vibrational mode shapes of the plate. The study aims to analyze the various factors that influence damage detection performance, such as the plate mode shape analyzed, the fidelity of the smoothing mesh used, SEA hyper-parameters, damage location, measurement noise, and proximity of the strain sensors to the damage site.

**Figure 2:**
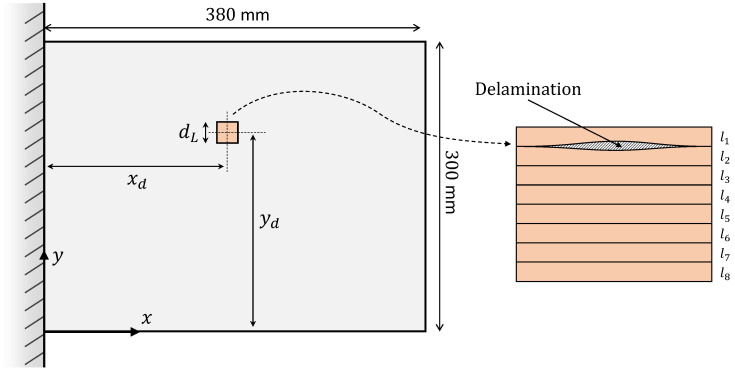
Illustration of the cantilevered composite plate with the parameters used to describe the delamination size and location.

The carbon–epoxy composite plate used for the study (shown in Figure 2) has a length L=380 mm, width W=300 mm, thickness 2t=2 mm, and is built of eight equal-thickness layers with a symmetric material layup (0/45/–45/90)${}_{s}$. The lamina material properties are provided in Table 1. The plate is initially modelled with a delamination of size $d_{L}=20$ mm at the interface of the first two layers ($l_{1}$–$l_{2}$), and located at $\left(x_{d},y_{d}\right)$ = (155,195) mm. The effect of the delamination position is studied later on in this section.

In the absence of an experimental plate model, a high-fidelity FE model (developed in ABAQUS) is used to study the mechanical behavior of the damaged plate. The FE strains are used as inputs for the iFEM analysis, and the FE displacements are the reference for iFEM comparisons. The plate is modeled using 4560 S4R elements, a four-node shell element in ABAQUS based on the first-order shear deformation theory (FSDT) with a bi-linear displacement field and reduced integration. The FE mesh chosen ensures a relatively fine discretization at the damaged region, with the delaminated area meshed using 16 shell elements. The delamination is modeled using the approach outlined by Ju et al. [54], where the upper and lower layers are meshed separately and the nodes are allowed to vibrate independently. The continuity of the displacements and rotations is satisfied at the delamination junctions. Numerous works in literature deal with predicting the mechanical behavior of damaged composite plates. Some interesting works include the modeling of the buckling behavior of normal or functionally-graded laminates with ply cracks [55,56]. Interested readers are encouraged to refer to these works for further details. The iFEM model uses the same discretization scheme and is modeled using the four-node inverse shell iQS4 [25], with bi-quadratic displacement interpolations and bi-linear rotations. In contrast to the direct FE model, the inverse mesh is developed with no separation of element layers at the delamination site.

**Figure 3:**
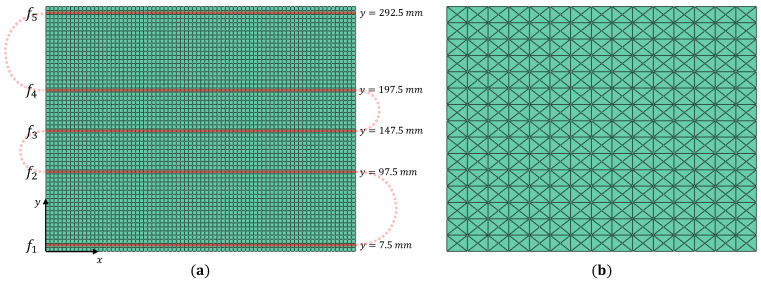
The discretization scheme for the plate: (**a**) FE/iFEM mesh with the fiber optic sensor paths shown, and (**b**) the 15×15 smoothing mesh using 900 SEA3 [51] elements.

The plate is instrumented with a 2 m long distributed fiber optic strain sensor, as shown in Figure 3. The sensor measures longitudinal strains ($\varepsilon_{xx}^{+}$) along five sensing lines ($f_{i}$, where i=1,...,5) on the plate top surface. These sensing lines are positioned along the center of the shell elements and measure the centroidal strains of each instrumented element. In its current state, sensing line $f_{4}$ overlaps the damage site on the plate. The proximity of the strain sensors to the delamination site is varied, and its consequent influence on damage detection is studied later in this section.

### 3.1. Vibration Mode Shape Reconstruction

**Figure 4:**
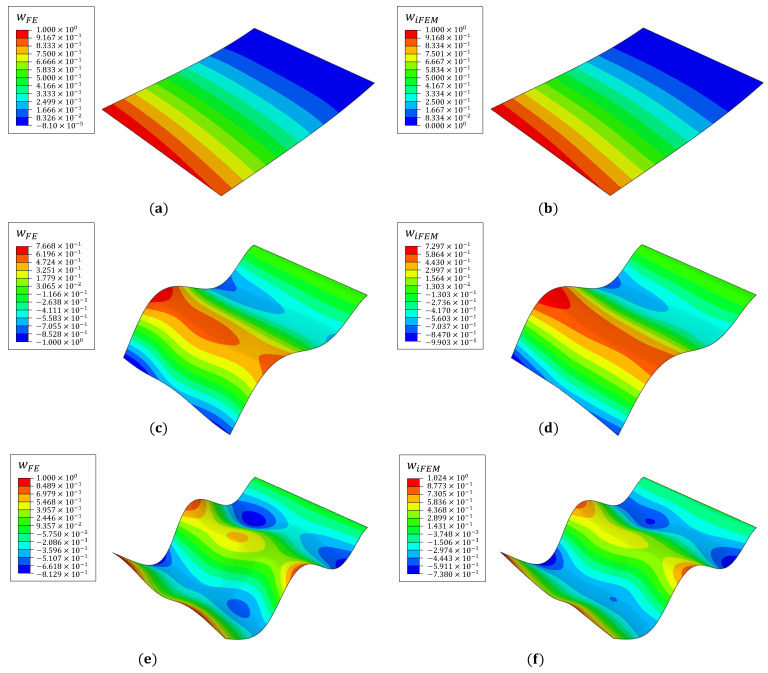
Reference FE and reconstructed iFEM mode shapes for (**a**,**b**) mode 1, (**c**,**d**) mode 8, and (**e**,**f**) mode 14.

Among the vibrational mode shapes, plate bending offers the highest potential for exciting the delamination and generating local strain perturbations. Also, as the fiber measures only longitudinal strains, iFEM accuracy for reconstructing the bending mode shapes is expected to be greater than for the in-plane or torsional modes. Alternatively, strain rosettes can be used instead of fiber optic sensors. In this case, the availability of triaxial strains is expected to produce more accurate results, with its associated difficulties of instrumentation and wiring. Given these practical considerations and the marginal gain in accuracy for the bending modes, fiber optic sensors are considered the ideal choice for the present study. The first three bending modes are considered: mode 1 at 17.15 Hz, mode 8 at 301.8 Hz, and mode 14 at 590.7 Hz. The iFEM analysis uses strains measured by all five sensing lines ($f_{1-5}$). For all elements with no strain measurements, a low value of weighting coefficient ($w=10^{-5}$) is used to formulate the corresponding element error functional. The contour plots of iFEM mode shapes compared against the reference FE results are shown in Figure 4.

With increasing mode number, the mode shapes are more complex, and the accuracy of the iFEM reconstruction decreases correspondingly. Results for the first mode are very accurate with a percentage error in nodal deflection, $w^{err}<0.5\%$ (where $w^{err}=100\times \left(w_{FE}-w_{iFEM}\right)/{\left(w_{FE}\right)}_{max}$). For the two higher modes, the tip errors are ∼10%, with lower errors ∼3% close to the fiber lines. Given the sensor configuration used, these results are considered reasonable. As the next stage of damage detection, Equation (2) is used to compute the damaged strain field over the plate top surface from the iFEM results.

### 3.2. Strain Smoothing Using the SEA

As the second stage of damage detection, the iFEM reconstructed damaged strain field is processed using the SEA to establish a healthy baseline. However, as the smoothing mesh and the value of hyper-parameters used affects the SEA results, they have to be optimally chosen a priori. To avoid biases associated with the choice of sensor positions, iFEM results from the full-field case are used for this specific study, i.e., when all inverse elements are instrumented with strain gauges oriented longitudinally.

**Figure 5:**
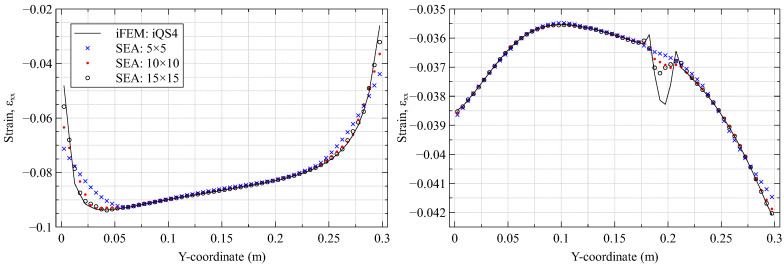
iFEM and SEA results (using α=1000) for various smoothing meshes compared along (**left**) the plate root (x=2.5 mm) and (**right**) across the delamination (x=162.5 mm).

Three smoothing mesh discretization schemes are used for this study, with 5, 10, and 15 subdivisions along the plate edge, respectively. The meshes use the three-node smoothing element SEA3 [52] arranged in a cross-diagonal pattern as shown in Figure 3. Similarly, among the hyper-parameters, only α is analyzed because it directly affects the continuity of the smoothed strains. As the analysis does not use noisy data, parameter β is set to a small value ($10^{-5}$). Two key features characterize an ideal SEA analysis: accurately capturing healthy or undamaged strains across the plate and filtering or smoothing the strain peaks and discontinuities near the damage site. The influence of the mesh on the SEA results is shown in Figure 5, where it is compared against the reference iFEM strains. The choice of an optimal mesh is a difficult balance between a low-fidelity mesh that smooths over both healthy and damaged strain peaks and a high-fidelity one that accurately captures both. The results of Figure 5 highlights the 15×15 mesh to be an ideal compromise.

**Figure 6:**
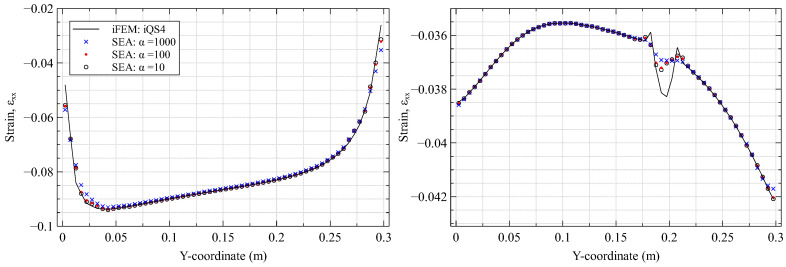
iFEM and SEA results (using 15 × 15 SEA3) for various values of α compared along (**left**) the plate root (x=2.5 mm) and (**right**) across the delamination (x=162.5 mm).

The influence of α on the SEA results is shown in Figure 6. Similar to the previous case, an optimal value of α should neither be too high, as $C^{1}$-continuity is rigidly enforced and the results smooth over both healthy and damaged strain peaks, nor too low to avoid piece-wise continuous results that accurately captures all peaks. The value of α=100 is considered an ideal compromise in this case. Based on the results of this study, a 15×15 smoothing composed of SEA3 elements and a parameter value of α=100 is used for all subsequent SEA analyses.

In conclusion, an optimal choice of the SEA parameters depend on the complexity of the strain field distribution, the noise level in the strain measurements, and the nature of the damage investigated. Hence, prior knowledge of these factors is required to make an optimal choice. Once these parameters are estimated for a specific SHM application, no further changes or corrections are required over the operational life of the structure. In this sense, it can be considered a necessary calibration of the method.

### 3.3. Delamination Detection

**Figure 7:**
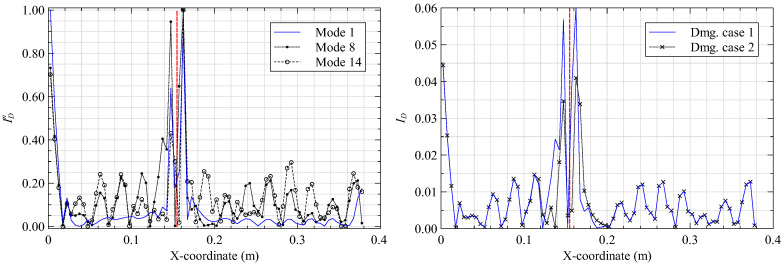
Damage index plots along y=197.5 mm for (**left**) damage case 1 and (**right**) mode 8 (the vertical red line indicates the damage site).

**Figure 8:**
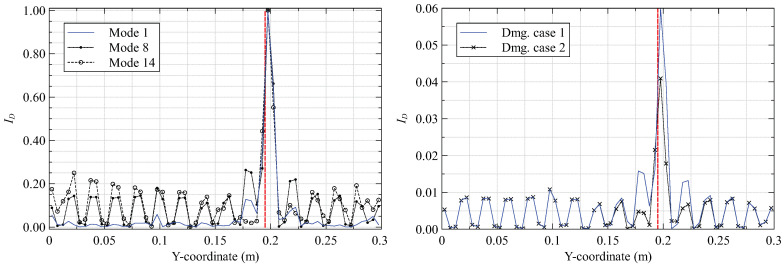
Damage index plots along x=162.5 mm for (**left**) damage case 1 and (**right**) mode 8.

**Figure 9:**
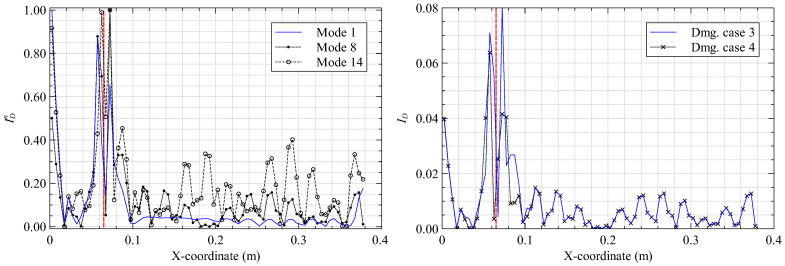
Damage index plots along y=197.5 mm for (**left**) damage case 3 and (**right**) mode 8.

**Figure 10:**
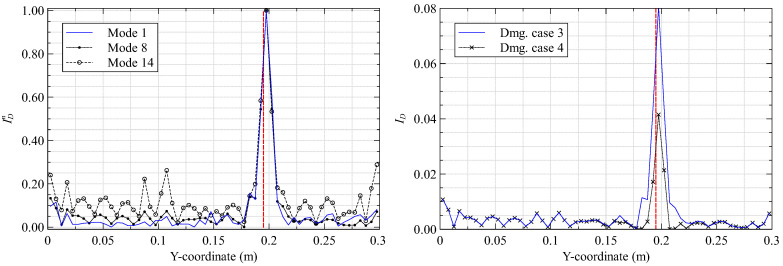
Damage index plots along x=72.5 mm for (**left**) damage case 3 and (**right**) mode 8.

The iFEM results of Section 3.1 are smoothed using the 15×15 SEA3 smoothing mesh to establish a healthy baseline. Subsequently, the damaged and baseline results are used to compute the damage index ($\kappa_{p}=\kappa_{x}$) based on Equation (10). Four different damage cases, varying the in-plane and through-thickness (inter-lamina) position of the delamination, are investigated to understand the effect of damage location on the proposed strategy. These cases are described in Table 2. Additionally, delamination detection as a function of the reconstructed vibrational mode shape is studied. The results from these studies are presented as line plots of $I_{D}$ along sections that intersect the damage location: x=162.5 mm and y=197.5 mm for cases 1 and 2, and x=72.5 mm and y=197.5 mm for cases 3 and 4. These results are shown in Figure 7, Figure 8, Figure 9 and Figure 10.

The results from damage cases 1 and 2 provide two key conclusions. First, all three bending modes excite the delamination, as indicated by the $I_{D}$ peaks at the damage site. It is also evident that the peaks corresponding to the higher modes are more prominent. This is due to the greater local excitation of the plate in the higher modes but at the cost of minor $I_{D}$ peaks at undamaged regions. The loss of iFEM accuracy with the increasing complexity of the higher modes explains these minor peaks, with probable misinterpretation of the damaged site as the obvious consequence. Among the three modes investigated, mode 8 provides a good compromise between these considerations. A second conclusion regards the through-thickness position of the damage. Delamination close to the plate surface produces a greater curvature gradient, leading to higher strain perturbations measured by the sensors and a greater $I_{D}$ peak for damage case 1 over 2.

These conclusions are reinforced by the results for damage cases 3 and 4. They also provide further insight. As plate bending produces the highest curvatures close to the clamped end or at local deflection peaks, delamination close to the clamp produces a higher $I_{D}$ peak than those further away. This is evident when comparing the $I_{D}$ peaks in Figure 8 and Figure 10. A limitation is the erroneous damage index peaks observed close to the clamped end in Figure 7 and Figure 9. These peaks are related to the inability of the SEA to accurately smooth the high strain gradients close to the clamp (also seen in Figure 5). Using a finer smoothing mesh close to the clamp is one way of circumventing this issue.

**Figure 11:**
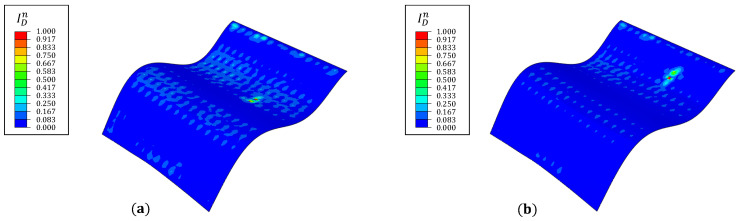
The damage index contour plots of mode 8 for damage case (**a**) 1 and (**b**) 3.

The results of the present study demonstrate the ability of the proposed method to detect delamination damages in vibrating composite plates. Furthermore, detection is easier for the higher modes when the delamination is close to the plate surface and to the clamped end. Contour plots of $I_{D}$ for the two best cases are shown in Figure 11.

### 3.4. Sensitivity to Measurement Noise

Despite the promise of previous results, they are obtained using numerical data measured by sensors instrumented over the damaged site. Practical scenarios are always less ideal with additional influences or difficulties, some of which are the primary focus of the present and following section.

**Figure 12:**
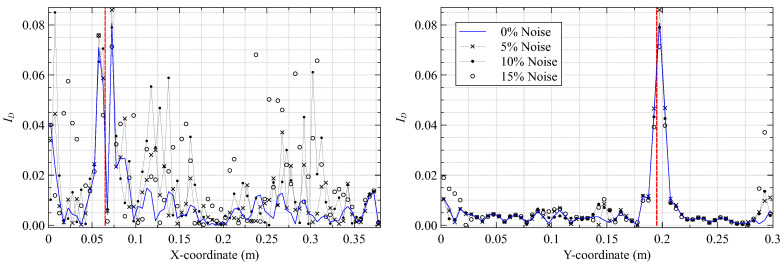
Damage index plots along (**left**) y=197.5 mm and (**right**) x=72.5 mm for different noise levels.

The numerical strains are artificially contaminated with random noise, added as a percentage of the strain magnitude. The aim of the study is to simulate sensor measurement, instrumentation, or experimental errors in a practical laboratory setting. The noise is introduced based on a Gaussian distribution with zero mean and the value of three standard deviations varying from 5% to 15%. The subject of the present study is the plate results for damaged case 3 and vibrating at mode 8. The noisy strains are the input for the iFEM analysis, and the subsequent damage detection steps are followed. The results for different noise levels are shown in Figure 12.

The damage detection results demonstrate good robustness up to a noise level of 10%, where the most prominent $I_{D}$ peak is still discernible at x=72.5 mm. At higher noise levels, this is no longer the case. Multiple peaks are evident at this stage, and the damage position can no longer be accurately identified. The surface strain perturbations produced by inter-laminar damage (like delamination) are lower than intra-laminar damage (like fiber/matrix cracking or lamina failure). Hence, using the current damage detection strategy, the former damage is expected to be more noise-sensitive.

An additional tool in such cases involves the SEA parameter β. Using a high value for β can suppress significant curvature discontinuities in the measured strains and filter the influence of random noise. However, a high value might also filter strain discontinuities associated with the damage. Additional studies are required to establish an ideal value.

### 3.5. Sensitivity to Sensor Location

**Figure 13:**
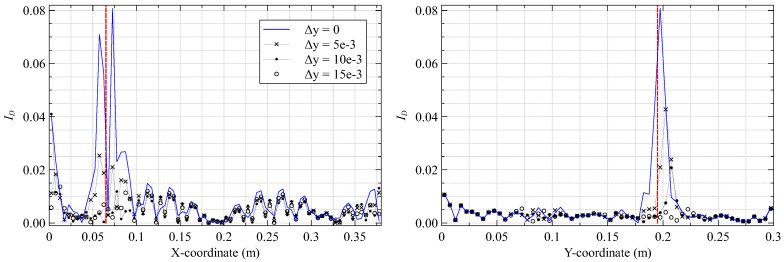
Damage index plots along (**left**) y=197.5 mm and (**right**) x=72.5 mm for different fiber locations.

A final study investigates the influence of sensor proximity to the delamination on damage detection. Previous iFEM analyses used in-situ strains based on Figure 3, where fiber line $f_{4}$ overlapped the delamination. Various new sensor configurations are simulated by shifting the position of fiber lines $f_{2}\left(y-\Delta y\right)$ and $f_{4}\left(y+\Delta y\right)$ symmetrically outward from the central line $f_{3}$. The parameter Δy represents the change in the vertical coordinate with respect to the original fiber position. Line $f_{4}$ is shifted from Δy=5 mm, where it is still within the delaminated region, to Δy=10−15 mm, where it is no longer in contact with it. The present study also investigates plate vibrating under mode 8 with a damage based on case 3. The results are shown in Figure 13.

The results show that the damage index peaks reduce in magnitude as the fiber is moved further away from the delamination site. This reduction is attributed to the highly localized nature of the damaged strain field produced by delamination and the low magnitude of strain perturbations measured by sensors placed further away. This study also reinforces another general conclusion of strain-based damage detection approaches, i.e., sensors must be instrumented close to the damage site to detect damage reliably. In this case, the optimal locations of strain sensors are a function of the damage type and the noise level in the measured strains. They are also influenced by the geometrical complexity of the structure, which is the subject of investigation in the next section.

## 4. Numerical Studies on a Composite Wing Box

**Figure 14:**
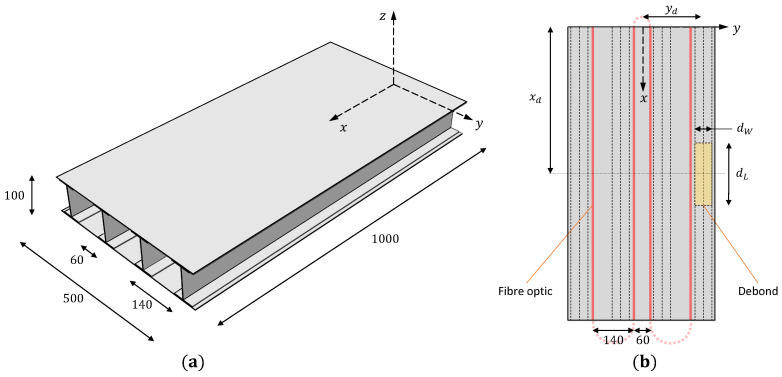
Illustration of the composite wing box showing the (**a**) geometric dimensions, (**b**) position of the fiber optic sensors, and the debonded area on the top skin (all dimensions are in mm).

The proposed strategy is also applied for monitoring damages in more complex aerospace structures. The structure analyzed in this case is a composite wing box composed of a top and bottom skin and stiffened with four longitudinal spars having an I cross-section. The skin, spar webs, and caps are 2 mm thick and have a symmetric material layup (0/45/–45/90)${}_{s}$ with lamina properties given in Table 1. The wing box is instrumented with distributed fiber optic strain sensors along four sensing lines per skin, with sensors placed on both the top and bottom skin surfaces. The geometric dimensions and sensor locations are given in Figure 14. The wing box is clamped at the root and loaded transversally at the tip by coupling the DOF of the tip nodes, essentially simulating a rib. A load of magnitude $F_{y}=1000$ N is applied. The present study investigates skin-spar debonding in the wing box. The debond is introduced between the upper skin and the outer spar, as shown in Figure 14. Three damage cases are analyzed, as described in Table 3, each varying the surface area ($d_{L}\times d_{W}$) of the debond. The fiber optic sensors are positioned to avoid overlapping the debonded region, with the most proximal sensing line on the upper skin located 10 mm away.

**Figure 15:**
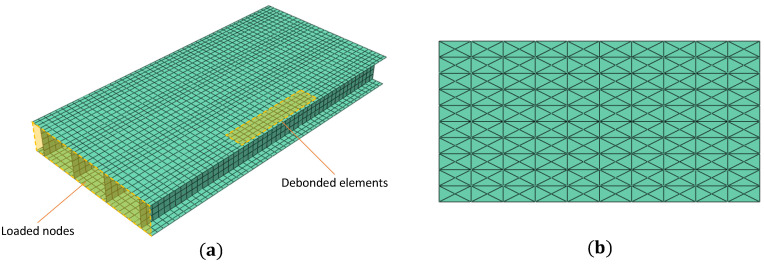
Element discretization for the (**a**) FE and inverse iFEM models, and (**b**) smoothing model of the wing box upper skin (10×10 SEA3 mesh).

A high-fidelity FE model of the wing box is developed in ABAQUS to simulate its mechanical behavior. The wing box is modeled using 5600 S4R elements, as shown in Figure 15. Over the debonded region, the skin and the spar cap are meshed separately using 64 shell elements, with displacement and rotation continuity maintained at the debond junctions [54]. An additional contact constraint is also introduced between skin and spar elements to avoid intersections during deformation. The FE strains are used as inputs for the iFEM analysis, and the FE displacements are the reference for comparisons. The iFEM model uses the same element discretization scheme as the direct model but employs the inverse shell element iQS4 [25]. Only the upper and lower skins of the wing box are assessed for debond detection, where the iFEM results are smoothed using a 10×10 SEA3 smoothing mesh shown in Figure 15.

### 4.1. Deformation Reconstruction

**Figure 16:**
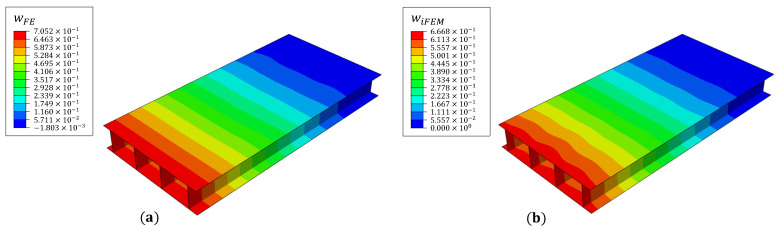
Deflection contours of (**a**) FE and (**b**) iFEM results for damage case 1 (units in mm).

The transverse tip load applied produces wing box bending and compression of the top skin. The compressive forces over the debond lead to localized in-plane strain or curvature peaks at the debond interfaces. The iFEM reconstructed deflection field for damage case 1 is compared with the reference FE results in Figure 16. The iFEM results report a maximum error of ∼9.6% close to the outer ends of the top or bottom skin (where no strain sensors are located) and ∼7% at the spar tips. These results, achieved only using longitudinal strain measurements, are deemed promising from the perspective of displacement or strain sensing and damage detection. Although optimizing the position of the fiber lines can further improve the accuracy of iFEM results [33], such investigations are not the focus of the present study.

### 4.2. Skin-Spar Debond Detection

**Figure 17:**
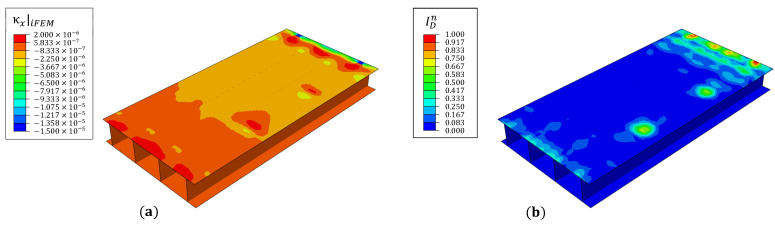
Contours of (**a**) iFEM reconstructed curvature and (**b**) damage index for case 1.

The iFEM reconstructed strains are processed using the SEA to establish a healthy baseline, and subsequently to compute the damage index ($\kappa_{p}=\kappa_{x}$). The contour plots of iFEM reconstructed curvature and the damage index over the top skin for damage case 1 are shown in Figure 17. The results indicate two damage index peaks at the debond junctions, corresponding to the locations of high curvature gradients. However, the results are also populated by erroneous peaks near the root and tip where the SEA does not accurately smooth undamaged strain gradients. These peaks negatively impact the lower limit of debond size that the proposed approach can reliably detect. As discussed in Section 3.3, adopting a fine smoothing mesh at these locations provides a strategy to eliminate the erroneous peaks.

**Figure 18:**
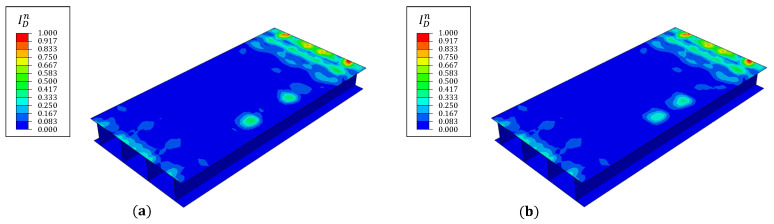
Contour plots of the damage index for damage case (**a**) 2 and (**b**) 3.

The 300 mm long debond described by damage case 1 represents significant damage (approximately 32% of the spar length), having a considerable influence on wing box strength. Smaller debond sizes, as described by cases 2 and 3, provide a more realistic picture of barely visible damages initiated by impacts on the structure by external projectiles. The damage index contour plots for these cases are shown in Figure 18 where, despite the smaller damage region, $I_{D}$ peaks corresponding to the debond junctions are seen. The relative magnitude of the peaks is also lower and is explained by the lower curvature gradients at the site. These results aid in demonstrating the viability of the proposed approach for delamination or debond detection. The key requirement for the proposed approach is that internal damages should produce a measurable change in the surface strain field of the structure. If the above condition is satisfied, the nature of the damage (intra- or inter-layer) should not be an impediment to damage detection. When analyzing intra-layer damages, fiber breakage is expected to have a more pronounced effect on surface strains than matrix cracking and fiber-matrix interface debonding, and hence should be more easily detectable. This does directly imply the improbability of detecting matrix cracking and interface debonding, but rather, further studies are required to reach a more accurate conclusion.

iFEM is independent of the material properties or operational conditions of the structure and it is unaffected by environmental factors as well. However, as strain is the physical quantity measured, environmental factors that influence strain can negatively affect the results obtained. Global temperature or humidity variations (affecting the whole structure) will not affect the proposed approach, while local variations can be problematic. Perhaps, the principal effect is local strain changes due to temperature variations. Local thermal strain changes might be misconstrued as being due to damages, leading to an erroneous displacement reconstruction and damage diagnosis. However, strategies exist to counter this limitation, such as the analytical modeling of the strain–temperature relationship or the use of temperature-compensated fiber optic strain sensors to simultaneously measure both elastic and thermal strain contributions. This latter solution is perhaps the more viable strategy. Furthermore, evaluating the stability or reliability of the damage detection approach requires a multi-parameter analysis involving all factors that influence the method. Metrics such as the probability of detection (POD) is useful in this regard to form an objective assessment of damage detection performance

Aside from its capabilities as an effective damage detection tool, the accuracy of the iFEM in reconstructing the displacements demonstrates its potential for simultaneous strain or stress sensing. In such cases, damage diagnosis can be combined with real-time prognosis or predictions of delamination or debond evolution based on the strain or stress time histories experienced by the structure. Developing such an integrated monitoring approach for all possible composite layups and damage or failure scenarios is a challenging problem and is the focus of future investigations.

## 5. Conclusions

This work presented a damage detection strategy based on the inverse Finite Element Method for detecting and monitoring intra- or inter-lamina damages in composite structures. The iFEM is initially used to reconstruct structural displacements and strains from surface strain-sensor measurements. Subsequently, the SEA is used to smooth the iFEM results and establish a real-time baseline. A comparison of the damaged and healthy data is used to predict the presence and location of the damage on the structure. The proposed approach is demonstrated numerically for delamination detection in a vibrating composite plate where the effect of damage location and operational conditions are studied. The results demonstrate that plates with delamination close to the plate surface, close to the clamped end, or when excited at higher frequencies produced the highest damaged surface strain perturbation. Consequently, this led to the best damage detection results. Additionally, the study demonstrated the method’s robustness to measurement noise and the depreciating damage detection performance with increased sensor distance from the damage site. The method’s viability for more complex structures is assessed through a numerical study on a composite wing box subjected to a concentrated tip load. Here, wing box failure due to skin-spar debonding initiated by compressive loads on the upper skin was studied. Using a curvature-based damage index and sensor measurements directly from the damage site, the results demonstrated reliable detection of debond interfaces across a range of damage sizes. A possible limitation is the erroneous damage index peaks at the clamped end, attributed to the improper smoothing of the healthy strains near the root. The choice of an optimal smoothing mesh can produce better results. These numerical studies were promising and demonstrate the proposed approach to be a reliable and robust SHM strategy requiring no information regarding the structure’s material properties, operational conditions, or healthy baseline state.

Despite the promising results, a key limitation is that strain sensors placed close to the damage site are required to ensure accurate predictions. Hence, it can be considered a local, rather than global, structural monitoring approach. Alternatively, strain pre-extrapolation or virtual sensing techniques can further reduce the number of sensors required without depreciating the method’s accuracy. Furthermore, the real-time strain or stress-sensing capability of the iFEM can be further exploited. From the perspective of damage prognosis and fatigue monitoring, the reconstructed strains and stresses can be used to make real-time estimates of the remaining operational life of the structure. Such investigations are the subject of future work.

## Figures and Tables

**Table 1:** The elastic material constants of the carbon fiber-reinforced epoxy composite lamina.

Young’s Modulus (GPa)		Shear Modulus (GPa)		Poisson’s Ratio		Density (kg/m^3^)
$E_{1}$	$E_{2}=E_{3}$	$G_{12}=G_{13}$	$G_{23}$	$\nu_{12}=\nu_{13}$	$\nu_{23}$	ρ
125	6.1	3.4	3.5	0.33	0.33	1446.2

**Table 2:** Details of the four delamination cases analyzed.

Case	$x_{d}$ (mm)	$y_{d}$ (mm)	$d_{L}$ (mm)	Lamina Interface
1	155	195	20	$l_{1}$–$l_{2}$
2	155	195	20	$l_{3}$–$l_{4}$
3	65	195	20	$l_{1}$–$l_{2}$
4	65	195	20	$l_{3}$–$l_{4}$

**Table 3:** The different skin-spar debond cases investigated.

Case	$x_{d}$ (mm)	$y_{d}$ (mm)	$d_{L}$ (mm)	$d_{W}$ (mm)
1	500	210	320	60
2	500	210	200	60
3	500	210	120	60

## Data Availability

The data presented in this study are available on request from the corresponding author.
